# Electrocardiographic assessment in obstructive sleep apnea: bridging pathophysiology and clinical practice

**DOI:** 10.3389/fphys.2026.1777406

**Published:** 2026-04-07

**Authors:** Frederic Roche, Marie Roux, Karim Benali, Vincent Pichot

**Affiliations:** 1 Université Jean Monnet, Saint-Étienne, France; 2 Centre de Recherche en Neurosciences de Lyon, Bron, France; 3 Centre Hospitalier Universitaire de Saint-Etienne, Saint-Étienne, France; 4 SAnte INgenierie BIOlogie St-Etienne, St-Priest-en-Jarez, France

**Keywords:** Sleep apnea, electrocardiography, cardiovascular physiology, arrhythmia, autonomic nervous system, heart rate variability, QT interval, artificial intelligence

## Introduction

**FIGURE 1:**
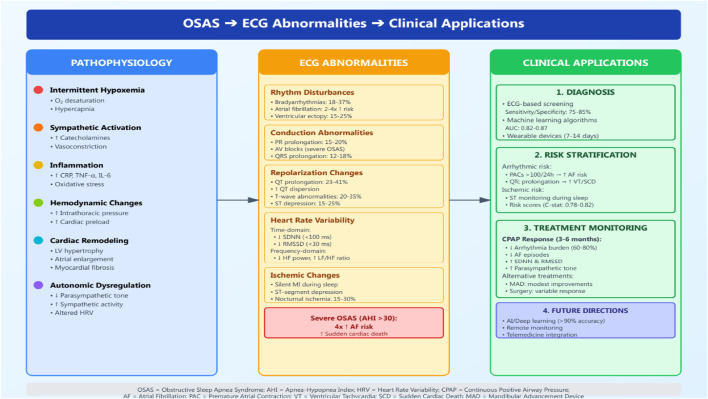
Comprehensive framework of ECG assessment in obstructive sleep apnea syndrome. The figure illustrates the pathophysiological mechanisms (left panel), associated ECG abnormalities (center panel), and clinical applications (right panel). Pathophysiological processes include intermittent hypoxemia, sympathetic activation, inflammation, hemodynamic changes, cardiac remodeling, and autonomic dysregulation. ECG abnormalities encompass rhythm disturbances (bradyarrhythmias, atrial fibrillation, ventricular ectopy), conduction abnormalities (PR and QRS prolongation), repolarization changes (QT prolongation, T-wave abnormalities, ST-segment depression), altered heart rate variability (reduced SDNN and RMSSD, decreased parasympathetic tone), and ischemic changes. Clinical applications include: (1) Diagnosis using ECG-based screening and machine learning algorithms with 75%–85% sensitivity/specificity; (2) Cardiovascular risk stratification for arrhythmic and ischemic risk; (3) Treatment monitoring showing CPAP response with 60%–80% reduction in arrhythmia burden and improvement in HRV parameters within 3–6 months; (4) Future directions including AI/deep learning applications with >90% accuracy and remote monitoring capabilities. AHI = apnea-hypopnea index; AF = atrial fibrillation; CPAP = continuous positive airway pressure; HRV = heart rate variability; OSAS = obstructive sleep apnea syndrome; PAC = premature atrial contraction; SCD = sudden cardiac death; VT = ventricular tachycardia.

Obstructive sleep apnea syndrome (OSAS) represents one of the most prevalent sleep-related breathing disorders, affecting approximately 50% of men and 23% of women aged 40 years or older when defined by an apnea-hypopnea index ≥15 events/h (Heinzer et al., 2015). Beyond its impact on sleep architecture and daytime function, OSAS has emerged as a significant cardiovascular risk factor, with mounting evidence demonstrating associations with hypertension, coronary artery disease, heart failure, and cardiac arrhythmias (Javaheri et al., 2024). The repetitive cycles of upper airway obstruction, intermittent hypoxemia, and arousal-mediated sympathetic activation create a unique pathophysiological milieu that profoundly affects cardiac electrophysiology. While polysomnography remains the diagnostic gold standard for OSAS, electrocardiographic (ECG) monitoring offers complementary insights that extend beyond traditional sleep parameters. The heart’s electrical signature of OSAS reflects complex autonomic, hemodynamic, oxidative stress and inflammatory processes that contribute to cardiovascular morbidity. Recent technological advances in automated ECG analysis, artificial intelligence (AI), and wearable monitoring have renewed interest in leveraging cardiac electrical signals for OSAS screening, risk stratification, and treatment monitoring (Figure 1).

**FIGURE 2:**
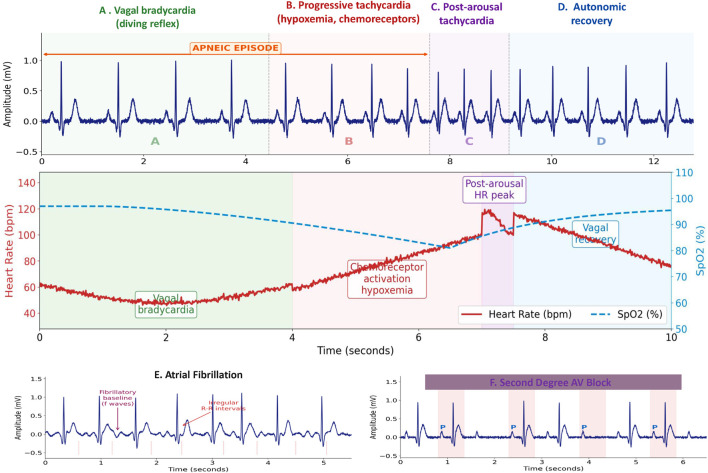
Representative ECG patterns in obstructive sleep apnea. Upper panel: Cyclical variation of heart rate (CVHR) during an obstructive apnea event showing the four autonomic phases: **(A)** bradycardia driven by vagal activation (diving reflex); **(B)** progressive tachycardia during hypoxemic phase (chemoreceptor activation); **(C)** post-arousal sympathetic peak tachycardia; **(D)** autonomic recovery and normalization of heart rate after resumption of breathing. Middle panels: **(E)** Atrial fibrillation pattern characterized by absent P waves, irregular R-R intervals, and fibrillatory baseline; **(F)** second-degree AV block (Mobitz II) with regular P waves and dropped QRS complexes, representative of severe nocturnal bradyarrhythmia in OSAS. Lower panel: Autonomic time-course of heart rate (red) and oxygen saturation (blue, dashed) dynamics across a full apneic cycle. CVHR = cyclical variation of heart rate; AF = atrial fibrillation; AV = atrioventricular; SpO2 = oxygen saturation.

This Opinion article synthesizes current evidence on ECG applications in OSAS management, examines the physiological mechanisms underlying cardiac electrical abnormalities, and proposes an integrated framework for incorporating ECG assessment into clinical practice. We argue that ECG monitoring represents an underused tool that can enhance cardiovascular risk assessment in OSAS patients and provide objective markers of treatment efficacy (Figure 2).

The evidence presented in this article was identified through a structured narrative review of the literature. We searched PubMed, MEDLINE, and EMBASE databases for publications up to December 2025 using the following key terms in various combinations: 'obstructive sleep apnea', 'electrocardiography', 'ECG', 'heart rate variability', 'arrhythmia', 'atrial fibrillation', 'QT interval', 'CPAP', 'autonomic nervous system', and 'artificial intelligence'. We prioritized original research articles, systematic reviews, meta-analyses, and major society guidelines published in peer-reviewed journals. Reference lists of identified articles were additionally screened for relevant studies not captured by the primary search. Inclusion was guided by methodological quality, clinical relevance, and recency, with emphasis on large cohort studies, randomized controlled trials, and validated risk scores. Studies were excluded if they lacked a control group, had sample sizes below 50 participants without compensating methodological strengths, or if their primary focus was pediatric populations.

## Pathophysiological mechanisms linking OSAS to cardiac electrical abnormalities

### Autonomic dysregulation and heart rate variability

The autonomic nervous system serves as a critical intermediary between respiratory events and cardiac electrical activity in OSAS. Repetitive apneic episodes trigger profound autonomic oscillations, with enhanced parasympathetic tone during respiratory efforts, hypoxemia-induced sympathetic surges followed by arousal-mediated parasympathetic withdrawal. These oscillations persist beyond sleep periods, contributing to sustained cardiovascular stress during wakefulness.

A hallmark electrophysiological feature of OSAS is the cyclical variation of heart rate (CVHR), a distinctive pattern observable on single-lead ECG recordings. During an obstructive apnea, the initial phase is characterized by bradycardia driven by the activation of pulmonary stretch receptors and enhanced vagal tone—the so-called 'diving reflex' response to hypoxemia and hypercapnia. As the apneic episode progresses, accumulating hypoxemia and hypercapnia progressively stimulate peripheral chemoreceptors (carotid bodies), generating a sympatho-excitatory surge that accelerates heart rate. At the moment of arousal and resumption of ventilation, a secondary tachycardia ensues due to sympathetic activation, followed by a vagally-mediated deceleration as breathing resumes. This cyclical pattern—bradycardia during apnea, tachycardia on arousal, then normalization—produces a characteristic oscillation in R-R intervals detectable on standard Holter or 30-s ECG strips. The amplitude and periodicity of CVHR correlate with AHI severity (r ≈ 0.65–0.75) and can achieve a sensitivity of 74% and specificity of 87% for moderate-to-severe OSAS when analyzed algorithmically from a single-lead ECG (Guilleminault et al., 1983; Sankari et al., 2017). From a prognostic standpoint, the persistence of CVHR despite CPAP therapy identifies patients with residual autonomic impairment and incomplete cardiovascular protection, underscoring its value as a dynamic treatment marker beyond simple AHI normalization.

Heart rate variability (HRV) analysis provides a broader non-invasive window into autonomic function. OSAS patients consistently demonstrate reduced daily time-domain parameters (SDNN, RMSSD) and altered frequency-domain indices, with decreased high-frequency (HF) power reflecting diminished parasympathetic tone and increased low-frequency to high-frequency (LF/HF) ratio indicating sympathovagal imbalance (Qin et al., 2021). Recent studies have confirmed that the magnitude of HRV reduction correlates with OSAS severity and predicts cardiovascular outcomes, positioning HRV as both a diagnostic marker and potential prognostic tool (Ucak et al., 2024).

It is increasingly recognized that OSAS is a heterogeneous syndrome with distinct physiological phenotypes that differentially modulate autonomic and ECG responses. Patients with high hypoxic burden—characterized by prolonged and severe oxygen desaturation per apneic event—demonstrate more pronounced QT prolongation, greater HRV impairment, and higher cardiovascular risk than those with equivalent AHI but lower desaturation profiles. REM-predominant OSAS, which is more common in women and in older patients, generates discrete bursts of sympathetic activation during REM sleep that produce distinctive ECG patterns—including clusters of ventricular ectopy and abrupt HRV oscillations—that differ quantitatively from those of NREM-predominant disease. Sex differences further modulate cardiac electrical responses: women with OSAS exhibit attenuated CVHR amplitude and different sympathovagal adaptation patterns compared to men of equivalent disease severity, which may contribute to the lower diagnostic sensitivity of ECG-based screening algorithms in female populations. These phenotypic distinctions have direct implications for ECG-based risk assessment and partially explain inter-individual variability in cardiac electrical findings at equivalent AHI levels, underscoring the need for phenotype-aware diagnostic and monitoring approaches.

### Hemodynamic and inflammatory pathways

Upper airway obstruction generates large negative intrathoracic pressure swings, increasing venous return and cardiac transmural pressure. Simultaneously, intermittent hypoxemia activates chemoreceptors, triggering vasoconstriction and blood pressure surges that increase cardiac afterload. These repetitive hemodynamic stresses contribute to left ventricular hypertrophy and atrial remodeling, creating substrates for arrhythmias (Javaheri et al., 2024). Chronic intermittent hypoxemia also promotes low-grade systemic inflammation and oxidative stress. These mediators accelerate atherosclerosis, promote endothelial dysfunction, and facilitate myocardial fibrosis, all of which influence cardiac conduction and repolarization (Amen et al., 2024).

## Spectrum of ECG abnormalities in OSAS

### Rhythm disturbances

Bradyarrhythmias represent an important and often underrecognized manifestation of OSAS. Nocturnal sinus bradycardia, sinus pauses exceeding 2.5 s, and varying degrees of atrioventricular (AV) block occur in 18%–37% of OSAS patients, predominantly during apneic episodes when coordinated vagal surges and hypoxemia-related conduction slowing converge. The severity and frequency of bradyarrhythmic events scale with AHI and nocturnal oxygen desaturation index. In a subset of patients, high-degree AV block may precipitate syncope, representing a directly treatable consequence of unrecognized OSAS. The 2022 American Heart Association/Heart Rhythm Society Scientific Statement on Sleep-Disordered Breathing and Cardiac Arrhythmias explicitly recommends ECG rhythm assessment—ideally *via* overnight cardiac monitoring—in patients with OSAS presenting with unexplained syncope, documented bradyarrhythmia, or prior to pacemaker implantation, as CPAP therapy alone may reverse conduction abnormalities and obviate device therapy (Mehra et al., 2022).

Atrial fibrillation (AF) demonstrates particularly robust associations with OSAS. Recent evidence confirms that OSAS increases AF risk two to four fold, with the Sleep Heart Health Study documenting that severe OSAS (AHI >30) confers a 4-fold increased AF risk (Mehra et al., 2022). A recent study found AF odds 2.5 times higher in OSAS patients (Alshomrani et al., 2024). Proposed mechanisms include atrial stretch from increased venous return, autonomic imbalance, and inflammatory-mediated atrial remodeling (Linz et al., 2023). The bidirectional relationship between OSAS and AF is clinically pivotal: patients referred from cardiology for AF evaluation or catheter ablation planning should be systematically screened for OSAS, given AHA/ACC guideline recommendations that OSAS identification and treatment may reduce AF recurrence (Mehra et al., 2022; Ndakotsu et al., 2024). Conversely, new-onset AF detected on Holter monitoring in a sleep clinic patient should prompt formal cardiac rhythm evaluation and cardiology co-management.

Ventricular arrhythmias, while less common than atrial arrhythmias, occur with increased frequency in severe OSAS. Complex ventricular ectopy and non-sustained ventricular tachycardia have been reported in 15%–25% of patients during sleep studies, with occurrence linked to hypoxemia severity and sympathetic activation.

### QT interval abnormalities and ventricular repolarization

QT interval abnormalities represent particularly concerning ECG findings in OSAS given their association with ventricular arrhythmias and sudden cardiac death. QT prolongation occurs in 23%–41% of patients with moderate-to-severe OSAS. A 2021 study demonstrated significant prolongation of Tp-e interval, Tp-e/QT ratio, and Tp-e/QTc ratio across increasing OSAS severity categories, with strong positive correlations between AHI and these repolarization parameters (Karacop and Karacop, 2021). Recent evidence shows that QTc prolongation in OSAS patients is associated with multiple cardiovascular risk factors including older age, female gender, higher BMI, and comorbidities such as diabetes mellitus and hypertension (Shi and Jiang, 2020). A pro-QTc risk score for mortality stratification demonstrated that higher scores independently predicted increased mortality risk across all OSAS severity categories (Memon et al., 2023). QT variability, reflecting beat-to-beat variations in ventricular repolarization, shows significant elevation during apneic episodes and correlates strongly with respiratory disturbance index severity. The mechanisms underlying QT abnormalities likely involve autonomic imbalance, intermittent hypoxemia-induced ion channel dysfunction, electrolyte disturbances, and sympathoadrenal activation with elevated catecholamines. Notably, CPAP therapy has been shown to improve QT parameters, suggesting reversibility of these repolarization abnormalities with effective treatment. T-wave abnormalities and ST-segment depression during sleep occur in 20%–35% and 15%–25% of OSAS patients, respectively, typically coinciding with apneic episodes and oxygen desaturation.

## Diagnostic applications of ECG in OSAS

### ECG-based screening algorithms

The accessibility and low cost of ECG monitoring make it an attractive screening tool for OSAS, particularly in settings where polysomnography access is limited. Recent developments in automated ECG analysis leverage machine learning and deep learning algorithms to detect OSAS-related patterns in heart rate dynamics, HRV parameters, and respiratory-related cardiac oscillations. A comprehensive meta-analysis evaluated the diagnostic accuracy of machine learning and deep learning algorithms for detecting sleep apnea from single-lead ECG data (Hosseini et al., 2025). The analysis demonstrated that current ECG-based screening algorithms achieve pooled sensitivity and specificity of 75%–85% for detecting moderate-to-severe OSAS. Recent deep learning models have achieved even higher performance, with convolutional neural network architectures demonstrating accuracy exceeding 90%–97% (Bazoukis et al., 2023; Liu et al., 2024). Multi-modal approaches combining ECG with oxygen saturation signals achieve per-segment detection accuracy of 91.38% and per-recording accuracy of 96.08% (Gao et al., 2025). A recent study from Mayo Clinic developed a deep convolutional neural network model achieving an AUC of 0.80 for OSAS identification, with particularly strong performance in females (AUC: 0.82) compared to males (AUC: 0.73) (Covassin et al., 2025). Multi-night ECG recordings enhance diagnostic yield by capturing night-to-night variability. Patch-based monitors and wearable devices worn for 7–14 days demonstrate excellent correlation with polysomnography while offering improved patient acceptability and real-world representativeness.

These performance metrics, however, warrant critical appraisal before clinical translation. The majority of published AI models were developed and evaluated on retrospective, single-center or consortium datasets, raising substantial concerns about external validity and overfitting. Dataset heterogeneity—encompassing differences in ECG acquisition protocols, patient demographics, OSAS severity distributions, and polysomnographic scoring criteria (notably hypopnea definitions) — complicates direct performance comparisons across studies. Many models have been trained and tested on overlapping or non-independent cohorts, which artificially inflates reported accuracy. Prospective, large-scale external validation in heterogeneous real-world populations—particularly in women, elderly patients, and those with significant comorbidities or polypharmacy—remains sparse, and published benchmark accuracies are rarely replicated outside their training environments. Until such validation is available, ECG-based AI algorithms should be regarded as promising research tools requiring further prospective validation rather than ready-to-deploy clinical diagnostics.

It is equally important to emphasize that ECG-based assessment is strictly complementary to polysomnography and does not replace it. Current major guidelines—including those from the American Academy of Sleep Medicine, the European Respiratory Society, and the American Heart Association—do not recommend ECG analysis as a standalone diagnostic modality for OSAS. ECG monitoring provides adjunctive cardiovascular information and may serve as a low-cost screening trigger in specific high-risk populations (e.g., patients with AF, unexplained syncope, or metabolic syndrome), but confirmed diagnosis and severity assessment of OSAS requires polysomnography or validated home sleep apnea testing. The clinical algorithms proposed in this article are designed to complement, not substitute, established diagnostic pathways.

### Cardiovascular risk stratification

Beyond OSAS diagnosis, ECG findings provide valuable prognostic information regarding cardiovascular risk. Frequent premature atrial contractions identify patients at heightened risk for AF development. QT prolongation and increased QT dispersion associate with ventricular arrhythmias and sudden cardiac death. The pro-QTc risk score demonstrates independent predictive value for mortality in OSAS patients (Memon et al., 2023).

Nocturnal heart rate and ECG-derived parameters have emerged as powerful independent predictors of cardiovascular morbidity and mortality in OSAS. Two landmark cohort studies have provided robust evidence in this regard. Sankari et al. (2019), analyzing data from the Wisconsin Sleep Cohort Study (n = 569, up to 15 years of follow-up), demonstrated that the nocturnal R-R interval dips index (RRDI) — reflecting beat-to-beat heart rate oscillations during sleep—was independently associated with composite cardiovascular events and mortality. Individuals in the highest RRDI category (≥28 dips/hour) had a 7.4-fold increased hazard for CVD incidence and mortality compared to those in the lowest category (HR 7.4, 95% CI 1.97–27.7, p = 0.003), independent of AHI, demographics, and comorbidities (Sankari et al., 2019). In a complementary approach, Azarbarzin et al. (2021) quantified the sleep apnea-specific pulse-rate response (ΔHR—the magnitude of heart rate increase following apneas and hypopneas) in the MESA (n = 1,395) and SHHS (n = 4,575) cohorts. Individuals with a high ΔHR were at significantly increased risk of non-fatal cardiovascular disease (adjusted HR 1.60, 95% CI 1.28–2.00), fatal CVD (adjusted HR 1.68, 95% CI 1.22–2.30), and all-cause mortality (adjusted HR 1.29, 95% CI 1.07–1.55), with the greatest risk amplification in those with concurrent high hypoxic burden. These findings collectively suggest that ECG-derived nocturnal heart rate metrics provide clinically actionable prognostic information beyond conventional AHI-based severity classification.

Risk stratification algorithms incorporating ECG parameters alongside OSAS severity and clinical variables outperform traditional cardiovascular risk scores in predicting adverse events. Ambulatory ST-segment monitoring during sleep reveals ischemic episodes in 15%–30% of OSAS patients, many of which occur silently without recognized anginal symptoms. Detection of nocturnal ischemia may prompt earlier coronary artery evaluation and revascularization in high-risk patients.

## Treatment monitoring with ECG

### CPAP therapy response

Continuous positive airway pressure (CPAP) represents the first-line treatment for moderate-to-severe OSAS, and ECG monitoring provides objective markers of therapeutic response. Studies consistently demonstrate significant improvements in most ECG parameters within 3–6 months of effective CPAP therapy. Nocturnal arrhythmia burden decreases by 60%–80% in adherent patients, with particular reductions in bradyarrhythmic episodes. AF burden shows substantial reduction with effective CPAP therapy, especially in patients with good adherence (Sánchez-de-la-Torre et al., 2023). QT parameters also improve with CPAP therapy, with studies demonstrating normalization of QTc intervals and reduction in QT dispersion.

Importantly, some ECG improvements may occur acutely with CPAP application. Optimal CPAP pressure significantly normalized R-R interval dip indices and reduced heart rate changes associated with non-apneic respiratory events, with RRI returning to baseline levels once resistive respiratory load was abolished (Sankari et al., 2017). This acute normalization reflects the immediate restoration of sympathovagal balance and elimination of hypoxemia-related autonomic excitation, even before structural cardiac remodeling can occur. This acute response has a practical clinical implication: the absence of short-term ECG improvement despite apparent CPAP adherence may signal suboptimal therapy (residual apneas, mask leak, or insufficient pressure) warranting re-titration.

HRV parameters consistently improve with CPAP therapy, with SDNN and RMSSD demonstrating significant increases within 6–12 weeks of treatment initiation. Greater improvements occur in patients with higher CPAP adherence, suggesting a dose-response relationship between treatment intensity and autonomic recovery. The magnitude of HRV improvement correlates with cardiovascular outcome benefits, positioning HRV monitoring as a potential tool for treatment optimization and adherence assessment (Qin et al., 2021).

### Alternative treatments

Surgical and device-based interventions targeting upper airway patency also demonstrate ECG improvements, though typically of lesser magnitude compared to CPAP therapy. A randomized controlled substudy of the STAR trial (Stimulation Therapy for Apnea Reduction) by Dedhia et al. (2019) demonstrated that hypoglossal nerve stimulation therapy in patients with moderate-to-severe OSAS produced significant improvements in SDNN and low-frequency HRV power across all sleep stages at 12 months, with the degree of HRV improvement correlating with AHI reduction achieved (Dedhia et al., 2019). Notably, a 1-week withdrawal of the stimulation therapy did not reverse the HRV improvements, suggesting durable autonomic remodeling. These observations support the concept that upper airway stabilization—regardless of the modality used—is the principal driver of autonomic cardiac recovery.

Weight loss deserves particular attention as a modifiable treatment strategy with direct cardiovascular electrophysiological benefits. Obesity independently impairs HRV, prolongs QTc, and promotes atrial remodeling; OSAS amplifies these effects synergistically. Sustained weight reduction of ≥10% body weight in obese OSAS patients significantly reduces AHI (by 20%–30%) and produces parallel ECG improvements including normalization of QTc, reduction in premature atrial contractions, and restoration of HRV parameters toward normal ranges. The cardiovascular ECG benefits of weight loss appear to operate through dual mechanisms: direct improvement in metabolic and inflammatory milieu (reducing adipokine-related myocardial stress) and OSAS-specific benefits from reduced upper airway fat deposition. Bariatric surgery, producing the largest and most durable weight reduction, has been associated with the most substantial ECG improvements, including reductions in AF burden and ventricular ectopy comparable to those seen with CPAP. Lifestyle-based interventions show proportionate ECG benefits aligned with the degree of weight loss achieved, further highlighting the importance of addressing obesity as a modifiable cardiovascular risk factor in OSAS management.

## Clinical implementation and future directions

### Integration into clinical practice

Professional societies increasingly recognize the value of ECG monitoring in OSAS management. The 2021 American Heart Association Scientific Statement recommends cardiac rhythm assessment in patients with moderate-to-severe OSAS or significant cardiovascular comorbidities (Yeghiazarians et al., 2021). Successful integration requires standardized protocols for ECG acquisition, analysis, and interpretation in the context of OSAS.

We propose a clinically grounded, guideline-aligned tiered approach to ECG utilization in OSAS that reflects real-world patient flow. In practice, ECG assessment in OSAS typically begins in two clinical contexts: (1) the sleep clinic, where patients are referred for suspected OSAS, and (2) the cardiology clinic, where patients with established arrhythmias—most commonly AF or unexplained bradycardia—are referred for sleep assessment prior to cardiac intervention. In the first context, the initial evaluation at the clinic visit centers on cardiovascular risk factor assessment (hypertension, diabetes, obesity, prior arrhythmia history, use of QT-prolonging medications). During the diagnostic sleep study, a single-lead ECG channel is routinely acquired during in-laboratory polysomnography (PSG), providing cardiac rhythm data throughout the night. In home sleep apnea testing (HSAT), pulse rate recording serves as a surrogate, though its resolution is inferior to dedicated ECG. When PSG reveals significant arrhythmias—AF, bradyarrhythmia, frequent ventricular ectopy, or concerning QT changes—a formal 12-lead ECG and/or 24–48 h Holter monitor is warranted as a second-tier evaluation. For high-risk patients with recurrent syncope, undocumented palpitations, or elevated sudden cardiac death risk, continuous ECG monitoring with implantable loop recorders provides long-term surveillance. In the second clinical context—the patient referred from cardiology—AHA guidelines recommend that OSAS be identified and treated prior to AF ablation, cardioversion, or antiarrhythmic therapy initiation, as OSAS treatment may itself resolve or significantly reduce arrhythmia burden (Mehra et al., 2022; Yeghiazarians et al., 2021).

## Technological advances and research priorities

Artificial intelligence and deep learning applications represent the Frontier of ECG-based OSAS assessment. The 2025 meta-analysis confirmed that machine learning algorithms analyzing raw ECG signals achieve diagnostic accuracy of 75%–85%, with the most advanced deep learning models exceeding 90%–97% (Hosseini et al., 2025; Bazoukis et al., 2023). Wearable devices offer a particularly promising avenue for bridging the gap between in-laboratory diagnostics and community-level surveillance. Consumer-grade smartwatches and medical-grade patch monitors worn for 7–14 days capture night-to-night variability in cardiac rhythm and HRV, providing a more representative picture of OSAS cardiovascular impact than a single PSG night. In patients initiating CPAP therapy, wearable ECG platforms can track HRV and arrhythmia burden trajectories over weeks to months, providing objective compliance-correlated treatment response data. Alerts generated by wearable AI algorithms for new-onset AF, significant QTc prolongation, or CVHR recurrence could facilitate earlier clinical intervention. Integration with telemedicine platforms creates a seamless loop from remote monitoring to clinical decision-making.

Future research should prioritize validation of ECG-based algorithms across diverse populations, including different age groups, ethnicities, and comorbidity profiles. Large-scale prospective studies are needed to establish whether ECG-guided risk stratification and treatment monitoring improve cardiovascular outcomes compared to standard care. Standardization of HRV and QT measurement protocols and establishment of population-specific reference values will enhance clinical utility.

## Discussion

The integration of ECG assessment into OSAS management represents a logical evolution in our understanding of sleep-disordered breathing as a multisystem disorder with profound cardiovascular implications. ECG monitoring offers several advantages: widespread availability, low cost, ease of implementation, and ability to provide continuous long-term surveillance. Unlike polysomnography, which captures a snapshot of a single night, ECG-based approaches can monitor night-to-night variability and assess treatment response over extended periods.

However, several important limitations must be acknowledged. First, ECG findings lack specificity for OSAS: bradyarrhythmias, QT prolongation, and HRV reduction are also observed in heart failure, hypothyroidism, coronary artery disease, and various cardiomyopathies, complicating attribution to OSAS alone. Second, cardiac medications exert profound effects on virtually all ECG parameters used for OSAS evaluation. Beta-blockers suppress HR and attenuate CVHR detection; QT-prolonging drugs (antiarrhythmics, certain antibiotics, antihistamines, antipsychotics) may independently extend QTc, making attribution to OSAS challenging. Stimulants, alcohol, and recreational substances alter sympathovagal balance and HRV in ways that mimic or exacerbate OSAS-related changes. A comprehensive medication and substance use history is therefore essential before interpreting ECG-derived autonomic metrics. Third, signal quality and artifact represent significant practical limitations in sleep ECG monitoring. Movement artifacts, electrode displacement during positional changes, and diaphoresis reduce signal fidelity, particularly during REM sleep—the stage with the highest sympathetic activity and arrhythmia burden. Many automated algorithms have been validated primarily on high-quality research-grade recordings and may underperform with routine clinical data. Fourth, most existing ECG-based OSAS algorithms have been developed and validated predominantly in middle-aged, predominantly male, Caucasian populations, restricting generalizability to women, elderly patients, and non-Caucasian ethnicities.

The greatest clinical value of ECG in OSAS may lie not in diagnosis *per se*, but in cardiovascular risk stratification and treatment monitoring. Identifying OSAS patients with high arrhythmia burden, prolonged QT intervals, increased QT variability, or nocturnal ischemia enables targeted interventions that may prevent adverse cardiovascular events. The pro-QTc risk score demonstrates how ECG parameters can be integrated into clinical decision-making tools for mortality risk stratification (Memon et al., 2023). Demonstrating ECG improvements with CPAP therapy provides objective evidence of treatment efficacy beyond subjective symptom improvement, potentially enhancing adherence.

The emerging capabilities of artificial intelligence and wearable technology promise to democratize access to cardiac monitoring and enable population-scale OSAS screening. Machine learning approaches achieve clinically meaningful diagnostic accuracy (Hosseini et al., 2025), with the most advanced models approaching or exceeding 90%. However, successful implementation requires careful attention to algorithm validation across diverse populations, data security, and clinical workflow integration. Overreliance on automated interpretations without appropriate clinical context could lead to overdiagnosis and unnecessary interventions.

Collaboration between sleep medicine specialists, cardiologists, and primary care physicians is essential for optimizing ECG utilization in OSAS. Sleep physicians should recognize cardiac electrical abnormalities that warrant cardiology referral, while cardiologists should maintain awareness of OSAS as a reversible contributor to arrhythmias and ischemia. Primary care physicians, who manage most OSAS patients, require education on interpreting ECG findings in the context of sleep-disordered breathing.

In conclusion, electrocardiographic assessment provides a valuable, accessible tool for enhancing cardiovascular risk evaluation in OSAS patients. It serves as a complementary modality to polysomnography and is not currently recommended as a standalone diagnostic tool by major sleep medicine or cardiology guidelines. ECG monitoring complements traditional sleep studies by providing insights into cardiac health and treatment response. As technology advances and our understanding deepens, ECG-based approaches will likely play an expanding role in personalized OSAS management, ultimately contributing to improved cardiovascular outcomes in this high-risk population.
